# Supplementary material for: Omics-based Investigation of Diet-induced Obesity Synergized with HBx, Src, and p53 Mutation Accelerating Hepatocarcinogenesis in Zebrafish Model
Source: Cancers (Basel). 2019 Nov 28;11(12):1899. doi: 10.3390/cancers11121899 (PMC6966430; doi:10.3390/cancers11121899)
Supplement: Supplementary file 1 [file cancers-11-01899-s001.pdf]

cancers

Supplementary Figures

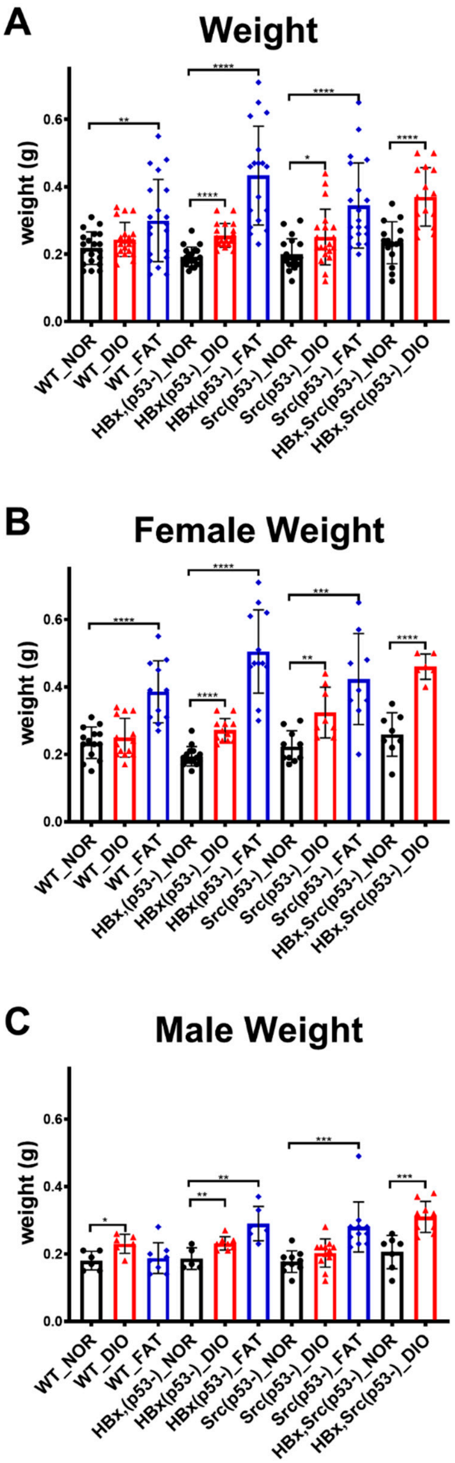

Figure S1. Body weight of four zebrafish models after feeding for eight weeks. (**A**) Average fish weight after eight weeks of normal diet (NOR), overfeeding (DIO), or high-fat diet (FAT). All zebrafish models had increased weight with DIO or FAT, and FAT fish weighed more than DIO fish.

(**B**) Average fish weight after 8 weeks of NOR, DIO, or FAT in female fish. (**C**) Average fish weight after 8 weeks of NOR, DIO, or FAT in male fish. The number of fish is 20 for each group.

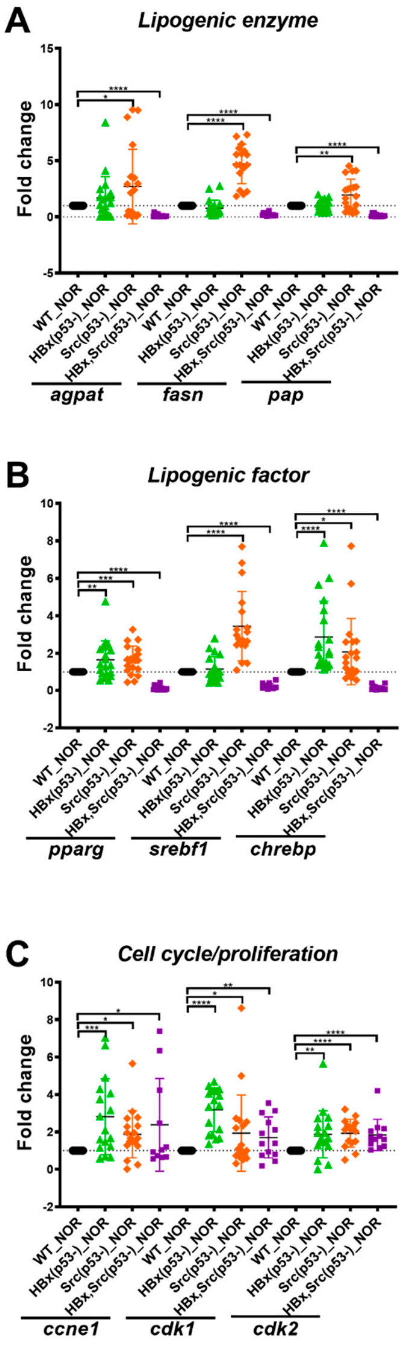

Figure S2. Expression of selected markers in various genetic background zebrafish with normal diet. Expression of (**A**) lipogenic enzymes, (**B**) lipogenic factors, and (**C**) cell cycle/proliferation related genes in WT, HBx(p53-), Src(p53-), and HBx,Src(p53-) fish under normal diet for two months starting at three months old (equivalent to five months old). Expression fold change compared to WT_NOR control. The number of fish is 20 for each group, and the number of experimental replicates for qPCR analysis is 3. Statistical analysis of results was performed using two-tailed Student's t test. Asterisks (*) represent level of significance: * p-value ≤ 0.05; ** p-value ≤ 0.01; *** p-value ≤ 0.001; **** p-value ≤ 0.0001.

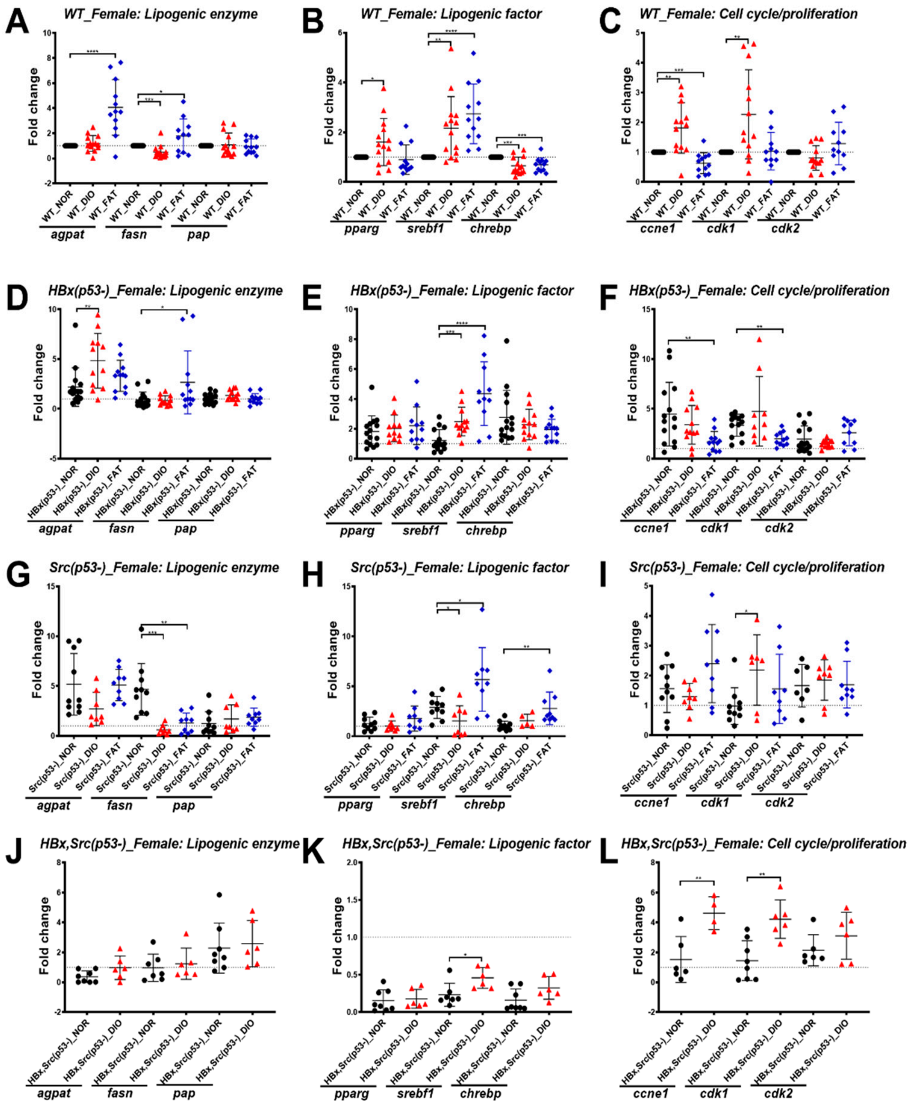

Figure S3. Expression of selected markers in various female zebrafish fed with different diets. Expressions of lipogenic enzymes (*agpat*, *fasn*, and *pap*), lipogenic factors (*pparg*, *srebf1*, and *chrebp*), and cell cycle/proliferation related genes (*ccne1*, *cdk1*, and *cdk2*) in female (**A–C**) WT, (**D–F**) HBx(p53-), (**G–I**) Src(p53-), and (**J–L**) HBx,Src(p53-) fish after eight weeks of normal diet (NOR), overfeeding (diet-induced obesity, DIO), or high-fat diet (FAT). Expression fold change compared to WT_NOR control. Each dot represented one fish in the graph, and the number of experimental replicates for qPCR analysis is 3. Statistical analysis of results was performed using two-tailed Student's t test. Asterisks (*) represent level of significance: * p-value ≤ 0.05; ** p-value ≤ 0.01; *** p-value ≤ 0.001; **** p-value ≤ 0.0001.

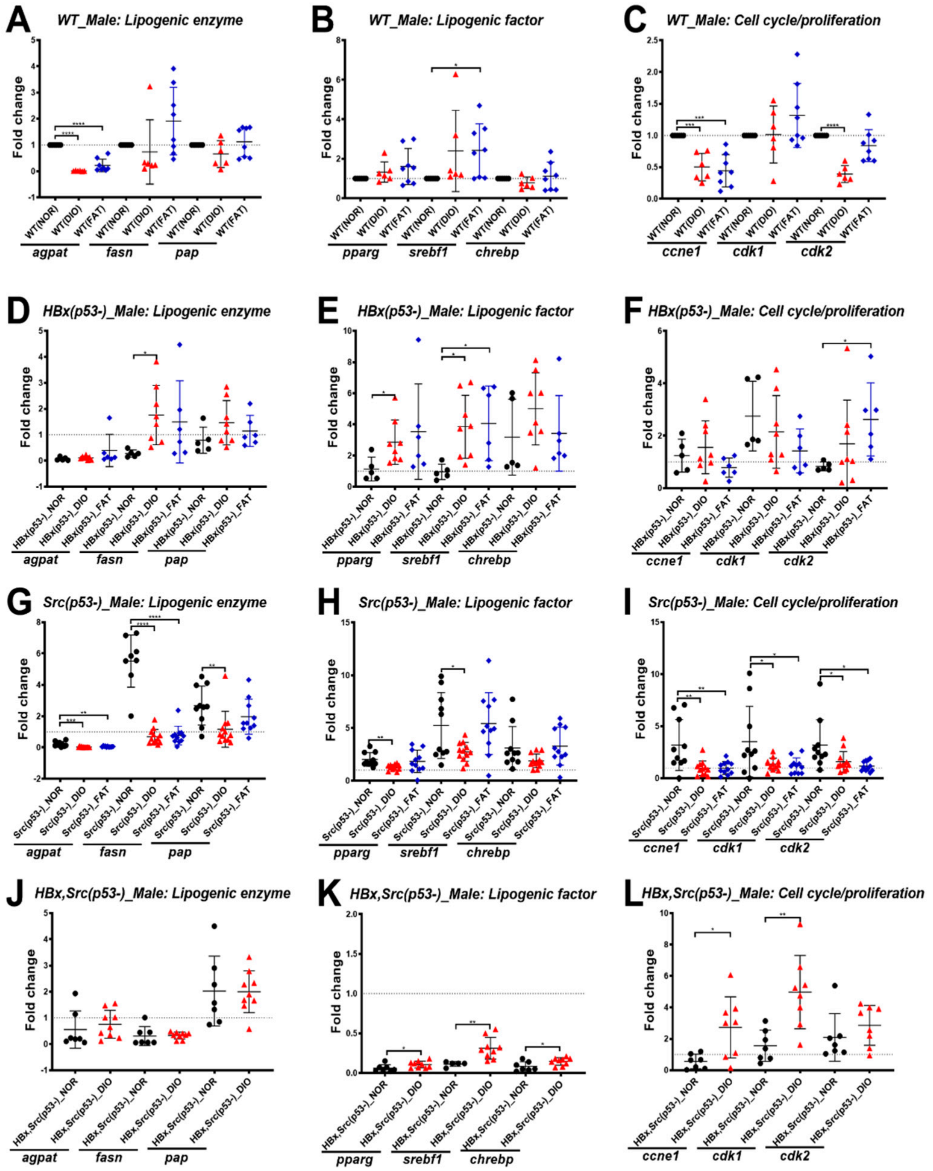

Figure S4. Expression of selected markers in various male zebrafish fed with different diets. Related gene expression of lipogenic enzymes (*agpat*, *fasn*, and *pap*), lipogenic factors (*pparg*, *srebf1*, and *chrebp*), and cell cycle/proliferation related genes (*ccne1*, *cdk1*, and *cdk2*) in male (**A–C**) WT, (**D–F**) HBx(p53-), (**G–I**) Src(p53-), and (**J–L**) HBx,Src(p53-) fish after eight weeks of normal diet (NOR), overfeeding (diet-induced obesity, DIO), or high-fat diet (FAT). Expression fold change compared to WT_NOR control. Each dot represented one fish in the graph, and the number of experimental replicates for qPCR analysis is 3. Statistical analysis of results was performed using two-tailed Student's t test. Asterisks (*) represent level of significance: * p-value ≤ 0.05; ** p-value ≤ 0.01; *** p-value ≤ 0.001; **** p-value ≤ 0.0001.

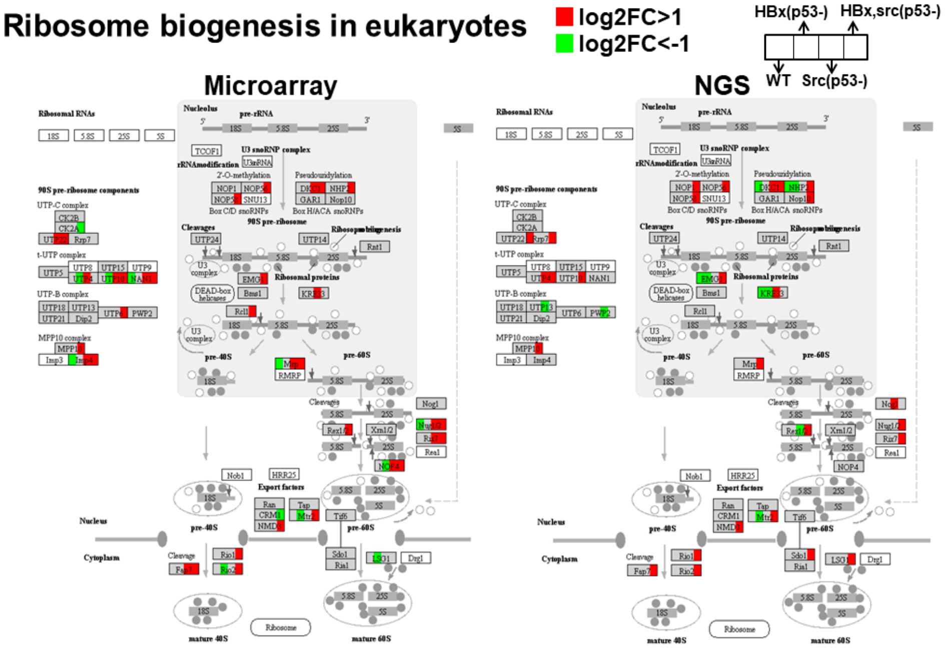

Figure S5. Expression values of selected differential expression genes for regulation of ribosome biogenesis pathway in various zebrafish fed with different diets. Four different genetic background of zebrafish were shown in order from left to right: WT, HBx(p53-), Src(p53-), and HBx,Src(p53-). Red and green represent up- and downregulated genes, respectively, based on KEGG database.

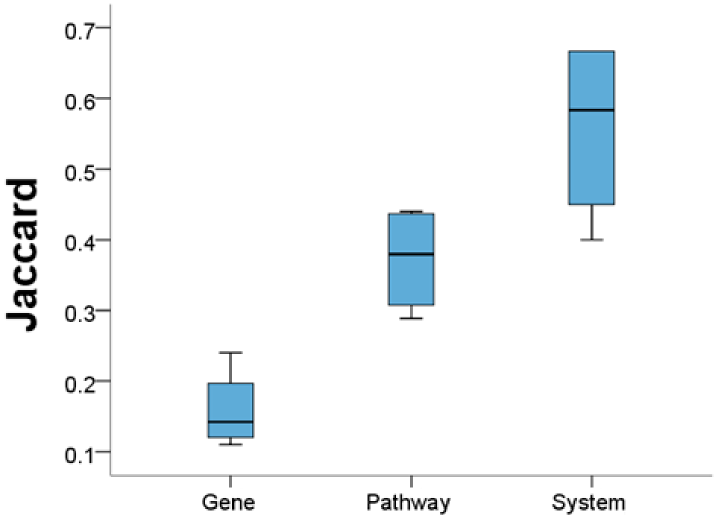

Figure S6. Jaccard index boxplot of microarray and NGS omics data for gene, pathway, and system levels. Jaccard median values of gene, pathway, and system levels are 0.14, 0.38, and 0.58, respectively.

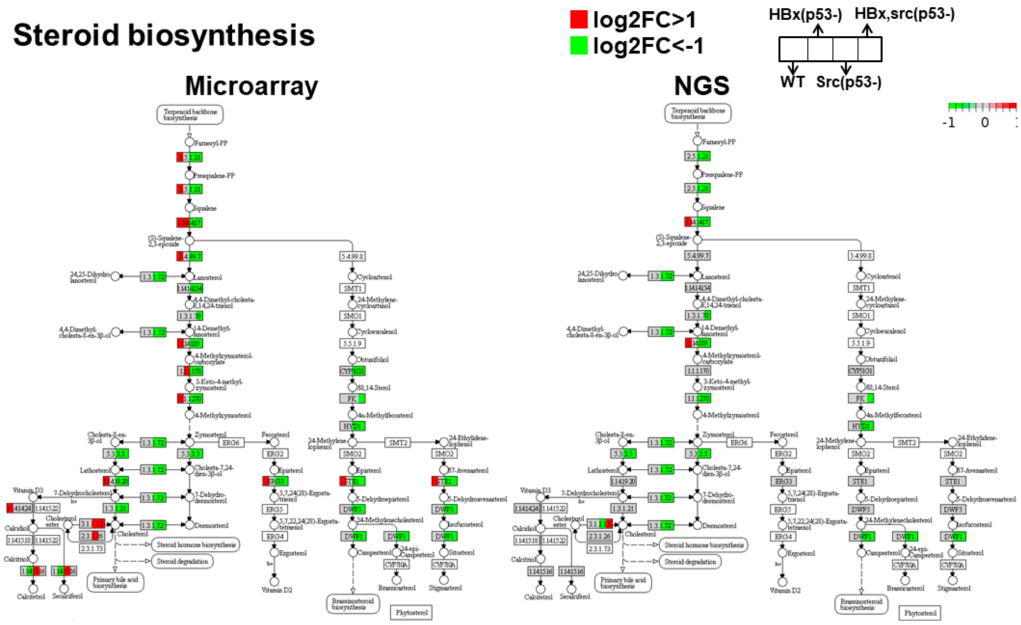

Figure S7. Expression values of selected differential expression genes for regulation of steroid biosynthesis pathway in various zebrafish fed with different diets. Four different genetic background of zebrafish were shown in order from left to right: WT, HBx(p53-), Src(p53-), and HBx,Src(p53-). Red and green represent up- and downregulated genes, respectively, based on KEGG database.

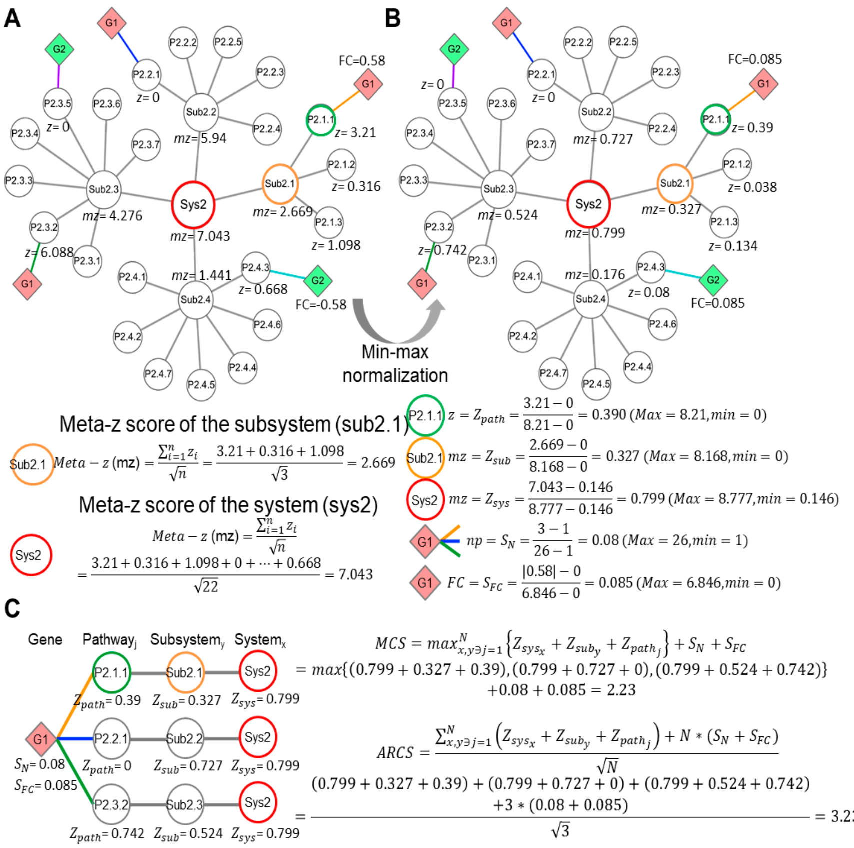

Figure S8. Schematic diagram of meta-z score and normalized meta-z score. Schematic diagram of calculating the (**A**) meta-z score, (**B**) normalized meta-z score (Z_{sys} and Z_{sub}), normalized z score (Z_{path}),

normalized values of number of involving pathways (S_N), and normalized values of $|log_2FC|$ (S_{FC}) of (**C**) MCS and ARCS.

Table S1. Experimental design of microarray and next-generation sequencing (NGS). There are 30 samples from four genetic fish models (wild-type (WT), HBx(p53-), Src(p53-), and HBx,Src(p53-)) treated with normal diet (NOR), overfeeding (diet-induced obesity, DIO), and high-fat diet (FAT).

**Platform**	Microarray			NGS		
**Time**	8 Weeks			8 Weeks		
Genotype \ Diet	NOR	DIO	FAT	NOR	DIO	FAT
WT	2	1	1	2	1	1
HBx(p53-)	1	1	1	2	1	1
Src(p53-)	1	1	1	2	1	1
HBx,Src(p53-)	2	2		2	2	

Table S2. The DEGs involved in insulin resistance pathway for four-type fish between normal and obesity diet (DIO and FAT) in microarray.

**WT**	**FC***	**HBx(p53-)**	**FC***	**Src(p53-)**	**FC***	**HBx,Src (p53-)**	**FC***
pik3cg	1.70	*socs3b*	2.07	*foxo1a*	4.69	*foxo1a*	3.01
nfkbiaa	1.54	*prkaa2*	1.70	*pck1*	4.52	*ppargc1a*	2.20
insra	1.09	*srebf1*	1.22	*ppargc1a*	3.00	*pck1*	1.83
prkag2a	1.07	*nfkbiaa*	1.12	*trib3*	2.02	*mgea5*	−1.06
mlxip	−1.02	*mapk8a*	1.04	*irs2a*	1.61	*g6pca.2*	−1.11
trib3	−1.03	*pck1*	−1.02	*pik3r3a*	1.50	*prkcq*	−1.22
pparab	−1.17	*rps6ka2*	−1.04	*prkag2a*	1.47	*creb3l2*	−1.28
LOC568883	−1.43	*creb3l2*	−1.11	*irs2b*	1.31	*mlxip*	−1.35
ppargc1a	−1.49	*LOC100334818*	−1.13	*g6pca.1*	1.27	*prkaa2*	−1.63
pck1	−1.68	*pik3ca*	−1.13	*cpt1ab*	1.26	*zgc:101540*	−1.64
irs2b	−1.69	*g6pca.1*	−1.21	*g6pca.2*	1.08	*g6pc3*	−1.65
		irs1	−1.24	*prkcda*	−1.13	*pygb*	−1.69
		foxo1a	−1.36	*prkaa2*	−1.43	*creb3l3a*	−1.72
		g6pca.2	−1.36	*prkcq*	−1.54		
		ppargc1a	−1.37	*ppp1r3b*	−2.02		
		prkcq	−1.46	*slc27a6*	−2.40		
		mgea5	−1.50				
		mlxip	−1.79				
		irs4a	−1.81				
		irs2a	−2.15				
		ppp1r3ca	−2.21				
		irs2b	−2.99				
		LOC568883	−3.04				
		slc27a6	−5.48				

* Values denote log_2 (fold change).

Table S3. The DEGs involved in insulin resistance pathway for four-type fish between normal and obesity diet (DIO and FAT) in NGS.

**WT**	**FC***	**HBx(p53-)**	**FC***	**Src(p53-)**	**FC***	**HBx,Src(p53-)**	**FC***
insrb	1.68	*prkcda*	1.64	*foxo1a*	3.68	*foxo1a*	2.96
pik3cg	1.50	*socs3b*	1.35	*g6pca.2*	2.19	*tnfrsf1a*	1.18

socs3b	1.40	*pik3r3b*	1.23	*irs2a*	1.69	*ppp1r3ca*	1.12
gys1	1.33	*rps6ka3b*	−1.05	*insrb*	1.03	*ins*	1.09
ppp1r3cb	1.26	*slc27a6*	−2.34	*zgc:77112*	−1.08	*ptpn11b*	1.03
tnfrsf1a	1.11	*ppp1r3ca*	−2.53	*prkag1*	−1.14	*nfkbiab*	−1.01
		irs2a	−2.60	*prkcq*	−1.21	*gfpt1*	−1.10
		g6pca.2	−3.05	*prkcda*	−1.79	*pik3cd*	−1.18
				pik3cg	−1.88	*pik3r3b*	−1.24
				slc27a6	−2.15	*slc27a1a*	−1.37
						creb3l3a	−1.56
						ikbkb	−1.62
						g6pca.2	−2.66

* Values denote log_2 (fold change).

Table S4. Top ranked 20 genes of MCS and their corresponding ranks of ARCS and FC for obesity.

Gene	MCS	Rank	ARCS	Rank	FC*	Rank
gck†	3.763	1	3.616	1	4.010	2
scd†	3.450	2	2.505	47	4.130	1
aldoab	3.399	3	3.096	4	2.839	8
hkdc1	3.308	4	3.311	2	1.965	19
pik3ca†	3.240	5	2.559	36	0.363	1676
acat2†	3.222	6	3.090	5	0.951	228
si:ch73-41h24.1	3.209	7	2.269	79	2.385	10
pik3cd†	3.209	8	2.527	42	0.234	2519
aldh2.1	3.207	9	2.907	9	1.055	171
pck1†	3.199	10	3.123	3	1.683	43
pla2g3	3.183	11	2.237	85	1.781	30
aldh7a1	3.172	12	2.895	10	0.745	471
sc5d	3.166	13	1.998	148	1.843	24
plcb3	3.165	14	2.026	139	0.715	516
aldh9a1a.1	3.164	15	2.864	11	0.875	304
pik3cb†	3.152	16	2.471	49	0.001	4649
fdft1†	3.148	17	1.980	154	1.768	31
cyp2r1	3.139	18	1.971	158	1.732	35
sqlea	3.138	19	1.970	159	1.728	36
aldh3a2b	3.131	20	2.830	12	0.738	485

* denotes absolute of log_2 (fold change). † denotes the obesity genes are recorded in DisGeNET.

Table S5. Top ranked 20 genes of MCS and their corresponding ranks of ARCS and FC for HCC.

Gene	MCS	Rank	ARCS	Rank	FC*	Rank
sqlea†	3.763	1	2.800	21	6.846	1
hmgcra†	3.450	2	2.203	108	5.420	2
mvda†	3.399	3	2.167	121	5.175	3
acat2†	3.308	4	3.599	1	2.116	66
aldh2.2†	3.240	5	3.117	5	2.167	57
zgc:153031	3.222	6	1.970	227	4.607	6
lss	3.209	7	2.496	39	4.767	4
fdps	3.209	8	2.103	153	4.738	5
hsd17b7	3.207	9	2.511	38	4.412	9
fdft1†	3.199	10	2.458	45	4.508	7
sc5d	3.183	11	2.446	47	4.428	8

pik3cd†	3.172	12	1.607	523	0.292	2644
cyp51	3.166	13	2.423	54	4.267	10
pik3ca†	3.165	14	1.583	546	0.130	3629
pik3cb†	3.164	15	1.581	548	0.117	3718
acox1†	3.152	16	2.690	30	2.844	23
dhcr24	3.148	17	2.406	60	4.151	11
ebp†	3.139	18	2.399	65	4.104	12
hkdc1	3.138	19	2.944	11	2.139	58
hadhab	3.131	20	3.321	2	1.167	432

* denotes absolute of log_2(fold change). † denotes the obesity genes are recorded in DisGeNET.

Table S6. Comparison of candidate genes selected from microarray and next-generation sequencing using two scoring methods for HBx(p53-) fish.

Gene	MIC_FC*	NGS_FC*	MIC_MCS
gck	2.77	3.96	3.438
abca1b	2.35	1.41	1.469
p4ha2	2.25	1.78	2.817
socs3b	2.07	1.35	2.500
fzd8a	1.95	1.36	1.718
inhbab	1.91	1.79	1.227
hkdc1	1.85	1.84	3.319
mpi	1.28	1.46	2.784
mtmr2	1.16	1.69	2.670
per2	1.10	2.32	0.784
axin2	1.10	1.84	1.491
mtmr8	1.08	1.50	2.655
aldoab	−5.80	−3.46	3.880
slc27a6	−5.48	−2.34	2.390
mycb	−3.21	−3.46	2.056
ppp1r3ca	−2.21	−2.53	2.357
irs2a	−2.15	−2.60	2.514
ldhba	−2.03	−2.24	3.190
mat2aa	−1.96	−2.51	2.808
prickle1a	−1.81	−1.60	1.614
glula	−1.65	−2.81	2.914
pim1	−1.56	−1.85	0.746
chac1	−1.55	−2.90	2.697
arg2	−1.48	−1.80	2.764
agxtb	−1.42	−2.08	2.835
g6pca.2	−1.36	−3.05	3.154
tob1b	−1.23	−1.98	1.183
hsd11b2	−1.18	−1.75	1.913
btg2	−1.16	−1.79	1.171
upp2	−1.14	−2.19	2.667
tdo2b	−1.08	−2.48	2.615
phykpl	−1.03	−1.60	2.607

* Values denote log_2(fold change).

Table S7. Comparison of candidate genes selected from microarray and next-generation sequencing using two scoring methods for Src(p53-) fish.

Gene	MIC_FC*	NGS_FC*	MIC_MCS
foxo1a	4.69	3.68	2.070
chac1	3.46	3.54	3.409
eif4ebp3l	2.69	2.48	2.009
glula	2.57	2.54	3.420
btg3	2.40	2.28	2.310
gbp	2.32	2.14	1.000
mknk2b	2.24	3.63	1.469
cdkn1a	2.08	3.47	1.525
eif4ebp3	1.98	2.63	1.857
polr1b	1.96	1.86	3.170
acss1	1.89	2.39	3.234
crata	1.78	1.51	0.659
eif4a2	1.78	2.37	1.815
gck	−3.66	−6.20	3.692
pmm2	−3.19	−4.00	3.392
itpa	−3.16	−2.00	3.386
fabp7a	−3.10	−3.14	1.369
acat2	−2.83	−3.17	3.715
scd	−2.77	−4.17	2.940
pim2	−2.67	−2.62	0.733
dhcr24	−2.48	−2.41	3.359
ebp	−2.40	−3.75	3.342
slc27a6	−2.40	−2.15	1.218
ckmt1	−2.28	−2.31	3.158
msmo1	−2.22	−2.32	3.305
pgd	−2.20	−3.16	3.220
tuba8l	−2.12	−2.35	1.083
mvda	−2.06	−2.60	3.110
mtmr2	−2.01	−2.02	3.139
mcoln1a	−1.92	−2.08	0.656
cmpk	−1.88	−2.23	3.112
psmd12	−1.86	−2.27	2.976

* Values denote $\log_2$(fold change).

Table S8. Comparison of candidate genes selected from microarray and next-generation sequencing using two scoring methods for HBx,Src(p53-) fish.

Gene	MIC_FC*	NGS_FC*	MIC_MCS	HCC_Positive
zgc:153031	4.61	3.33	3.616	0
upp2	2.89	3.94	3.366	0
tdo2b	2.65	3.25	3.291	0
pop5	2.65	2.51	2.954	0
asah1a	2.48	3.48	3.305	1
emg1	2.31	2.26	2.864	0
zgc:86811	2.14	2.22	3.256	0
mphosph10	2.13	2.16	2.838	0
gbp	2.03	2.05	0.521	0
pim2	1.93	2.48	0.282	0
phkg1a	1.88	2.15	0.609	0

foxo1a	1.87	2.96	0.941	1
dkc1	1.87	2.18	2.799	1
mphosph6	1.82	2.51	1.851	0
mknk2b	1.76	2.39	0.590	0
eif4ebp3l	1.70	2.31	2.340	0
sqlea	−6.85	−5.63	3.903	1
hmgcra	−5.42	−5.78	3.695	1
mvda	−5.18	−5.35	3.659	1
fdft1	−4.51	−4.85	3.562	1
hsd17b7	−4.41	−2.38	3.588	0
dhcr24	−4.15	−4.13	3.510	0
ebp	−4.10	−4.45	3.503	1
msmo1	−3.80	−4.31	3.458	0
elovl6	−3.33	−2.22	3.470	1
hspa5	−3.01	−3.64	2.544	1
elovl2	−3.00	−3.03	3.421	0
gatm	−2.91	−2.65	3.369	0
mvk	−2.88	−2.06	3.364	1
tm7sf2	−2.75	−3.11	3.306	1
mknk1	−2.65	−2.20	0.721	0
vps37b	−2.55	−2.76	0.724	0
calr3b	−2.41	−2.86	2.456	0
rdh12	−2.34	−2.23	3.246	1
tuba8l	−2.33	−2.75	0.836	0
rock2a	−2.31	−2.42	0.796	1
prdx1	−2.30	−2.26	1.033	1
aldoab	−2.28	−2.20	3.396	0
scd	−2.27	−2.34	2.803	1
hyou1	−2.27	−2.29	2.395	0
tuba8l4	−2.23	−2.20	0.820	0
mycb	−2.20	−2.48	0.745	1
hkdc1	−2.14	−2.61	3.496	0
pdia6	−2.14	−2.25	2.376	0
acat2	−2.12	−2.24	3.653	1
dlat	−2.11	−2.32	3.332	0
sesn3	−2.09	−2.47	0.421	0
psat1	−2.07	−2.78	3.326	0
ckmt1	−2.04	−2.35	3.201	0

* Values denote $\log_2$(fold change).

Table S9. Primer sequences for lipogenic factors/enzymes and cell cycle/division related genes.

Gene Name	Primer Name	Start	Sequence (5′ to 3′)	Accession Number	Size (bp)
**Lipogenic Factors**					
pparg	Q-*pparg*-F	513	GGTTTCATTACGGCGTTCAC	NM_131467.1	250
	Q-*pparg*-R	762	TGGTTCACGTCACTGGAGAA		
srebf1	Q-*srebf1*-F	2163	CATCCACATGGCTCTGAGTG	NM_001105129.1	250
	Q-*srebf1*-R	2412	CTCATCCACAAAGAAGCGGT		
chrebp	Q-*chrebp*-F-2	818	GGAGATGGACTCGCTCTTTG	XM_001338467	200
	Q-*chrebp*-R-2	513	GCAGAGGCTCAAAAGTGTCC		
**Lipogenic Enzymes**					

fasn	Q-*fasn*-F	7183	ATCTGTTCCTGTTCGATGGC	XM_682295	250
	Q-*fasn*-R	7432	AGCATATCTCGGCTGACGTT		
pap	Q-*pap*-F	976	CAGTTCTTCCTGATTGCTGC	XM_692415	250
	Q-*pap*-R	1225	TCCTCAAAGCTTAGTTCGGG		
agpat	Q-*agpat* -F	517	TTGGCGAAAAAGGAACTGTC	NM_212992	250
	Q-*agpat*-R	820	GGTGGTACTTGAGTTTGGGG		
**Cell Cycle/Division Related Genes**					
ccne1	Q-*ccne1*-F	371	TCCCGACACAGGTTACACAA	NM_130995.1	201
	Q-*ccne1*-R	571	TTGTCTTTTCCGAGCAGGTT		
cdk1	Q-*cdk1*-F	779	CTCTGGGGACCCCTAACAAT	NM_212564.2	200
	Q-*cdk1*-R	978	CGGATGTGTCATTGCTTGTC		
cdk2	Q-*cdk2*-F	794	CAGCTCTTCCGGATATTTCG	NM_213406.1	199
	Q-*cdk2*-R	992	CCGAGATCCTCTTGTTTGGA		

Table S10. Mapping of RNA-seq reads in 16 samples to reference genome (GRCz10/danRer10).

Sample	Clean Reads	Total Mapped Reads
WT_NOR20	11,605,217	10,354,175 (89.22%)
WT_NOR2	14,053,254	12,522,855 (89.11%)
WT_DIO16	13,408,586	12,014,093 (89.6%)
WT_FAT12	12,494,528	11,062,655 (88.54%)
HBx_NOR15	11,375,926	10,052,906 (88.37%)
HBx_NOR2	10,839,593	9,728,535 (89.75%)
HBx_DIO13	13,873,295	12,259,831 (88.37%)
HBx_FAT12	11,653,976	10,492,075 (90.03%)
Src_NOR5	12,073,502	10,895,128 (90.24%)
Src_NOR9	10,769,748	9,557,074 (88.74%)
Src_DIO11	14,319,836	12,791,909 (89.33%)
Src_FAT4	13,653,133	12,128,078 (88.83%)
Tri_NOR11	14,227,675	12,830,517 (90.18%)
Tri_NOR3	13,451,757	12,008,383 (89.27%)
Tri_DIO11	14,164,324	12,314,463 (86.94%)
Tri_DIO7	13,793,680	12,412,933 (89.99%)

© 2019 by the authors. Submitted for possible open access publication under the terms and conditions of the Creative Commons Attribution (CC BY) license (http://creativecommons.org/licenses/by/4.0/).
